# Supplementary material for: Guidance to best tools and practices for systematic reviews
Source: BMC Infect Dis. 2023 Jun 8;23:383. doi: 10.1186/s12879-023-08304-x (PMC10247272; doi:10.1186/s12879-023-08304-x)
Supplement: Supplementary file 4 — Additional file 5A. Illustrations of the GRADE approach. [file 12879_2023_8304_MOESM4_ESM.pdf]

Additional File 5A: Illustrations of the GRADE approach

Table AF5A-1: The outcome-centric approach in GRADE

Source	PICO question	Outcomes evaluated[a]	Outcome importance[b]	Systematic review	Recommendation formulation
				Certainty of a body of evidence[c]	Overall certainty of evidence[d]
Hanson 2019[1] and Crocket 2012[2]	Should lubiprostone be used in the management of opioid-induced constipation in patients with non-cancer pain?	Spontaneous bowel movement response	Critical	Low ⊕⊕◯◯	Low
		Reduction in severity of straining	Important	Moderate ⊕⊕⊕◯	
		Adverse effects leading to discontinuation of treatment	Important	Moderate ⊕⊕⊕◯	
Lantos 2021[3]	In patients following a high-risk tick bite, should prophylactic antibiotic therapy be used *versus* observation?	Clinical evidence of Lyme disease after treatment	Critical	Moderate ⊕⊕⊕◯	Moderate
		Seroconversion	Important	Low ⊕⊕◯◯	
		Serious adverse events	Important	Moderate ⊕⊕⊕◯	

[a]See original citations for a complete list of outcomes that are reported in the GRADE evidence profiles;
[b]Multiple stakeholders participate in defining which outcomes are critical and important. This is an early step in the process for developing clinical recommendations;
[c]Determined by consideration of factors that affect confidence in an estimate of effect.[4] See Table 5.1 in main text for the specific reasons for upgrading and downgrading the certainty of evidence;
[d]For recommendations, overall certainty across outcomes is determined by the lowest certainty of evidence for any outcome rated as critical.[5]

Table AF5A-2: Statement of conclusions on evidence certainty reached with and without application of GRADE[a]

**Topic of interest:** *Should treatment (X) be used to treat (condition) in (population)?*<br>**Critical outcomes:** *Quality of Life (QoL), significant adverse events (AEs)*<br>**Systematic review research question:** *Is treatment X more effective than usual care for improving QoL in (condition)?*	
**Without application of GRADE**	**With application of GRADE**
Treatment X compared to usual care leads to statistically significant improvements in QoL.	There is (*high, moderate, low, very low*) certainty evidence that Treatment X compared to usual care improves QoL in (population) with (condition).
Treatment X was not associated with significant AEs compared to usual care.	There is (*high, moderate, low, very low*) certainty evidence that Treatment X does not cause more frequent AEs compared to usual care in (population) with (condition).
There is sufficient evidence to suggest Treatment X over usual care for improving QoL in (condition).	There is (*high, moderate, low, very low*) certainty evidence that Treatment X is more effective for improving QoL compared to usual care in (population) with (condition).

[a]The example is a hypothetical systematic review. Adapted from Samuniak and colleagues.[6]

REFERENCES

1. Hanson B, Siddique SM, Scarlett Y, Sultan S. American Gastroenterological Association Institute technical review on the medical management of opioid-induced constipation. Gastroenterology. 2019;156(1):229-253.e5.
2. Crockett SD, Greer KB, Heidelbaugh JJ, Falck-Ytter Y, Hanson BJ, Sultan S. American Gastroenterological Association Institute guideline on the medical management of opioid-induced constipation. Gastroenterology. 2019;156(1):218–26.
3. Lantos PM, Rumbaugh J, Bockenstedt LK, Falck-Ytter YT, Aguero-Rosenfeld ME, Auwaerter PG, et al. Clinical practice guidelines by the Infectious Diseases Society of America (IDSA), American Academy of Neurology (AAN), and American College of Rheumatology (ACR): 2020 guidelines for the prevention, diagnosis and treatment of lyme disease. Clin Infect Dis. 2021;72(1):e1–48.
4. Guyatt G, Oxman AD, Sultan S, Brozek J, Glasziou P, Alonso-Coello P, et al. GRADE guidelines: 11. Making an overall rating of confidence in effect estimates for a single outcome and for all outcomes. J Clin Epidemiol. 2013;66(2):151–7.
5. Schünemann H, Brozek J, Guyatt G, Oxman A (Eds). Section 5.4: Overall quality of evidence. GRADE Handbook [internet]. GRADE; 2013 [cited 2022 Mar 25]. Available from: https://gdt.gradepro.org/app/handbook/handbook.html#h.lr8e9vq954a.
6. Samuniak D, Watts C, Cumpston M, Lasserson T, Livingstone N, Opiyo N. Common errors: a resource for Cochrane Editors. Cochrane; 2016 [cited 2022 Mar 5]. Available from: https://training.cochrane.org/common-errors.

Kolaski K, Logan LR, Ioannidis JPA. Guidance to best tools and practices for systematic reviews. BMC Infectious Diseases. 2023. Epub ahead of print. https://doi.org/10.1186/s12879-023-08304-x
